# Lifestyle behaviors in Swedish university students before and during the first six months of the COVID-19 pandemic: a cohort study

**DOI:** 10.1186/s12889-022-13553-7

**Published:** 2022-06-16

**Authors:** Kristina Larsson, Clara Onell, Klara Edlund, Henrik Källberg, Lena W. Holm, Tobias Sundberg, Eva Skillgate

**Affiliations:** 1 Department of Health Promotion Science, Sophiahemmet University, Stockholm, Sweden; 2 Unit of Intervention and Implementation Research On Worker Health, Institute of Environmental Medicine, Karolinska Institutet, Stockholm, Sweden

**Keywords:** COVID-19, Lifestyle behaviors, Physical activity, Meal habits, Alcohol, Tobacco, Substance use, Students, Longitudinal

## Abstract

**Background:**

Changes in Swedish university students’ lifestyle behaviors during the COVID-19 pandemic are unknown. This study aimed to assess physical activity, sitting time, meal frequency and risk substance use (alcohol, tobacco, and illicit use of drugs) in Swedish university students before and during the first six months of the COVID-19 pandemic, for all and stratified by age and sex.

**Methods:**

Data were obtained from the Sustainable University Life cohort study in which web-based surveys were sent to university students repeatedly for one year. Baseline assessment (before the pandemic) was between August 2019-March 2020, follow-up 1 (FU1) between March-June 2020, and follow-up 2 (FU2) between June–September 2020. Participants reported weekly minutes of physical activity, daily sitting hours, meal frequency by weekly intake of different meals, and motivation for eating irregularly, if so. Also, harmful use of alcohol, tobacco and illicit drugs was assessed. Population means and differences with 95% confidence intervals (95% CI) in lifestyle behaviors between time points were calculated with Generalized Estimating Equations.

**Results:**

1877 students (73% women, mean age 26.5 years) answered the baseline survey. Weekly exercise decreased by -5.7 min (95% CI: -10.0, -1.5) and -7.7 min (95% CI: -12.6, -2.8) between baseline and FU1 and FU2, respectively. Weekly daily activities increased by 5.6 min (95% CI: 0.3, 11.7) and 14.2 min (95% CI: 7.9, 20.5) between baseline and FU1 and FU2. Daily sitting time decreased by -1.4 h (95% CI: -1.7, -1.2) between baseline and FU2. Breakfast intake increased by 0.2 days per week (95% CI: 0.1, 0.3) between baseline and FU2. Lunch intake decreased by -0.2 days per week (95% CI: -0.2, -0.1) between baseline and FU1 and by -0.2 days per week (95% CI: -0.3, -0.0) between baseline and FU2. Dinner intake decreased by -0.1 days per week (95% CI: -0.2, -0.0) between baseline and both FU1 and FU2. Only minor differences in risk substance use were observed. Similar changes were observed in analyses stratified by age and sex.

**Conclusions:**

Lifestyle behaviors in Swedish university students slightly improved during the first six months of the COVID-19 pandemic compared to before.

**Trial registration:**

ClinicalTrials.gov, NCT04465435. 10/07/2020.

**Supplementary Information:**

The online version contains supplementary material available at 10.1186/s12889-022-13553-7.

## Background

Unhealthy lifestyle behaviors are public health challenges of global concern, contributing to mortality and burden of disease with implications for individual and societal health. In 2017, unhealthy lifestyle behaviors accounted for an estimated 37% of disability-adjusted life-years and over 23 million deaths [1]. In contrast, healthy lifestyle behaviors including high levels of physical activity, low levels of sitting time and healthy eating reduce the risk of mortality [2, 3], cardiovascular disease [4, 5], type 2 diabetes and several forms of cancer [6, 7]. Eating breakfast most days of the week is associated with better health [7–10] as well as a higher intake of fruits and vegetables [11]. Moreover, a low use of alcohol, tobacco and illicit drugs reduces the risk for both mortality and disability [1].

The outbreak of the COVID-19 pandemic became a global emergency in 2020. Social distancing and lockdowns may have had a substantial impact on our everyday lives regarding how we work, study, and move in society. On March 13, 2020, the spread of COVID-19 in Sweden entered a more intense phase [12], however, as opposed to most other countries, the Swedish government decided to implement recommendations for social distancing, as opposed to a full-scale lockdown of society [12]. On March 17, 2020, the Public Health Agency recommended all universities to cancel campus-based activities and move to remote learning on digital platforms [13]. University students have been particularly affected in their everyday lives given these adaptations, which also may have entailed changes in their lifestyle behaviors. Prior to the pandemic, we knew that university students spend more time sitting than the general young population [14]. Also, university students are more prone to unhealthy lifestyle behaviors such as insufficient physical activity and low intake of fruit, vegetables, and dietary fiber [15]. Students who eat breakfast report lower prevalence of depressive symptoms [16, 17] and higher academic achievements compared to students who do not have breakfast [18]. Further, American and British university students report higher levels of alcohol consumption compared to individuals in the same age who do not study [19, 20]. Swedish male university students have previously reported more physical activity compared to female students [21]. Also, Swedish male university students have reported a higher consumption of alcohol per drinking occasion compared to female students [22]. Beyond that, studies describing Swedish university students’ lifestyle behaviors are scarce.

Studies report mixed results regarding changes in university students’ lifestyle behaviors during the COVID-19 pandemic. Systematic literature reviews conclude that physical activity levels have decreased among university students in general, except among those who already met minimum recommendations before the pandemic [23, 24]. Changes in physical activity levels among university students during the pandemic do not only impact physical health but has also been shown to be associated with psychological distress [25]. Further, increased sitting time has been observed [26] but also increased self-reported physical activity [27]. Students with healthy and unhealthy food habits, respectively, before the pandemic have reported unchanged habits during the pandemic [27]. Moreover, a higher energy intake and frequency of snacking among students during the pandemic have been reported [28]. Food insecurity (i.e. low access to safe and nutritious food) is associated with physical and mental health problems [29], unhealthy food habits [30] and obesity [31], and has been reported among students during the pandemic [32, 33]. Furthermore, both increased and decreased use of tobacco, alcohol and cannabis have been reported [34–38].

It is to date unknown if Swedish university students´ lifestyle behaviors have changed during, compared to before, the COVID-19 pandemic. The primary aim of this study was to assess Swedish university students’ levels of physical activity, sitting time, meal frequency, alcohol use, tobacco use, and illicit use of drugs before and during the first six months of the COVID-19 pandemic. Furthermore, the aim was to assess potential differences in subgroups stratified by age and sex.

## Methods

This study aimed to assess lifestyle behaviors of Swedish university students before and during the first six months of the COVID-19 pandemic.

### Study design and setting

The study is based on the Sustainable UNiversity Life study (SUN-study; http://clinicaltrials.gov/ID: NCT04465435) [39], a prospective cohort aiming to identify modifiable risk- and prognostic factors for depression, anxiety, stress, and musculoskeletal pain in university students. Students enrolled at educational programs at universities in Stockholm County with at least one academic year left until graduation were eligible to participate. Recruitment to the SUN-study was ongoing between August 2019 and November 2020. Since the aim of this study was to compare lifestyle behaviors before and during the COVID-19 pandemic, only students enrolled between August 2019 and March 2020 were invited to participate (*n* = 6681).

### Data collection

The SUN-study was conducted through web-surveys. Students were recruited through in-class presentations, social media channels and information meetings at campus. Students at selected universities and education programs in Stockholm County were invited to answer a baseline survey focusing on factors of importance for mental- and musculoskeletal health, including lifestyle behaviors. Included participants received a follow-up survey every third month for one year.

To assess lifestyle behaviors before and during the first six months of the COVID-19 pandemic, three periods of measurement were specified. August 19, 2019 to March 13, 2020, was defined as the baseline assessment, before the COVID-19 pandemic, when all included participants filled out the survey. Thereafter, participants filled out a follow-up survey at two timepoints, defined as periods during the COVID-19 pandemic. Follow-up 1 (FU1) was between March 14, 2020, and June 15, 2020 and follow-up 2 (FU2) between June 16, 2020, and September 10, 2020. For enrollment in this study, participants had to answer at baseline as well as at FU1 and/or FU2.

### Variables

#### Physical activity

Physical activity was assessed with two questions from the National Board of Health and Welfare [40]. Participants were asked to report how many minutes, during a normal week, they spent exercising (e.g., running, fitness training, ball sports) and daily activities (e.g., walking, cycling, gardening), with categorical response alternatives. To convert the categorical response alternatives to a continuous scale, the middle value in each category was used. Combined physical activity was calculated as a sum of minutes per week in physical training and daily activities, in which minutes in physical training were doubled to account for intensity.

### Sitting time

Daily sitting time was assessed with one question about how many hours per day participants were sedentary [41] with categorical response alternatives. The middle value in each category was used to convert the categorical response alternatives to a continuous scale.

### Meal frequency

Participants were asked to report a weekly frequency of eating breakfast, lunch, dinner, meal between breakfast and lunch (Snack 1), meal between lunch and dinner (Snack 2), meal after dinner (Night snack) and meals other than those previously specified (referred to as Other meals) [42]. Also, participants were asked to state the reason for having irregular eating habits, if relevant. Meal frequency was presented as mean days per week of intake for each meal.

### Alcohol, smoking and substance use

The Alcohol, Smoking and Substance Involvement Screening Test (ASSIST) was used to assess harmful use of alcohol and tobacco as well as illicit (non-medical) use of drugs including cannabis, cocaine, amphetamine-type stimulants, inhalants, sedatives/sleeping pills, hallucinogens and opioids [43]. The ASSIST generates a score between 0–42 categorized as low (alcohol score 0–10, other substances score 0–3), moderate (alcohol score 11–26, other substances score 4–26) or high (above score 27) risk substance use.

### Statistics

Statistical analyses were conducted using R statistical system (version 1.2.5019). Generalized Estimating Equations (GEE) with an exchangeable correlation matrix were used. GEE is a population average approach appropriate when using repeated measurements and when data is not normally distributed. All lifestyle behaviors were analyzed as continuous variables. Separate models were conducted to assess overall mean differences for physical activity, sitting time, meal frequency and alcohol, smoking and substance use over the three periods. Analyses with the full group were adjusted for follow-up period, age, and sex. Subgroup analyses were conducted in which participants were stratified by sex (men/women, adjusted for follow-up period and age) and median age (≤ 25 years/ > 25 years, adjusted for follow-up period and sex).

### Sensitivity analyses

The number of participants analyzed at each period varied. Therefore, also complete case analyses were performed including only participants who participated in all three measurements. The complete case analyses assessed validity in terms of risk of attrition over time, which may have affected the results and thereby the conclusions. Results from the complete case analyses were compared with the results from the analysis with all participants.

## Results

**Fig. 1:**
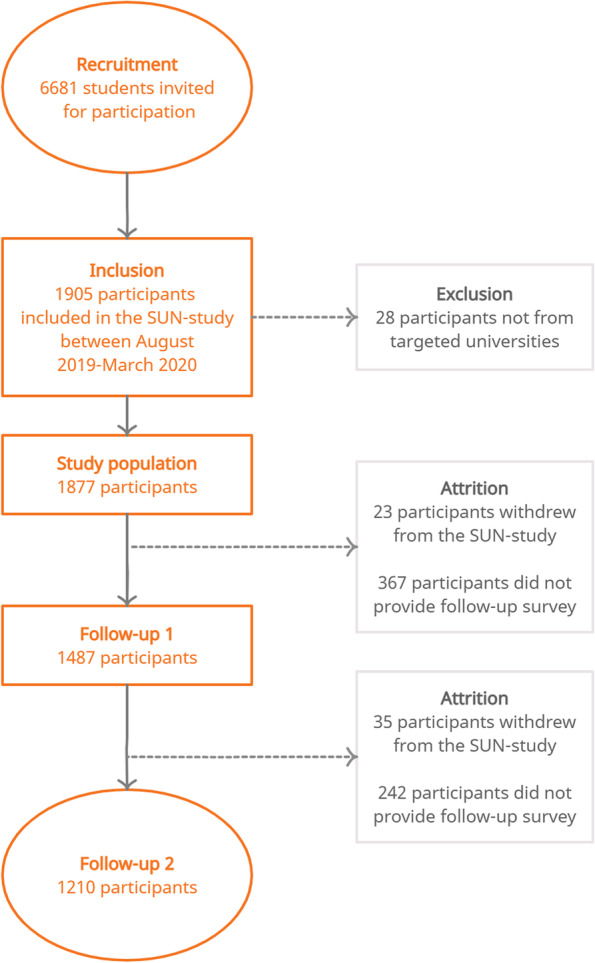
Flow-chart of participant inclusion and assessments

In total, 6681 students were invited to participate in the SUN-study before the outbreak of the COVID-19 pandemic in Sweden, and 1877 students (28%) enrolled. Flowchart of participant inclusion is presented in Fig. 1. Participant characteristics at baseline and FU2 are presented in Table 1.

**Table 1:** Participant characteristics at baseline and follow-up

	**Baseline (*n* = 1877)**	**Follow-up 2 (*n* = 1210)**
Mean age, years (SD)	26.5 (6.8)	27.0 (7.1)
Women, *n* (%)	1376 (73)	919 (76)
Children younger than 18 years, *n* (%)	291 (16)	205 (17)
Moved to study, *n* (%)	520 (28)	337 (28)
Field of study		
Health/medicine, *n* (%)	1675 (89)	1106 (91)
Other, *n* (%)	202 (11)	104 (9)
Civil status		
Unmarried/single, *n* (%)	867 (46)	534 (44)
Married/living with partner, *n* (%)	761 (41)	515 (43)
Living apart, *n* (%)	220 (12)	141 (12)
Divorced/ separated, *n* (%)	29 (2)	20 (2)
Living situation		
With partner/spouse, *n* (%)	752 (40)	510 (42)
Alone, *n* (%)	576 (31)	375 (31)
With parent/parents, *n* (%)	372 (20)	222 (18)
With friends, *n* (%)	128 (7)	77 (6)
In the student dormitory, *n* (%)	49 (3)	26 (2)
Poor sleep quality, *n* (%)	557 (30)	266 (22)
Moderate to extremely severe levels of stress symptoms^1^, *n* (%)	451 (24)	189 (16)

^1^Assessed with Depression, Anxiety and Stress Scale 21, score > 9 categorized as moderate symptom levels [44]

Mean time in minutes in physical activity (daily activities, exercise, and total physical activity) per week and hours spent sitting per day are presented in Table 2. Minutes in exercise decreased with -5.7 min (95% CI: -10.0, -1.5) and -7.7 min (95% CI: -12.6, -2.8), respectively, from baseline to FU1 and FU2 for the full group. Furthermore, daily activities increased with 5.6 min (95% CI: 0.3, 11.7) per week between baseline and FU1, and with 14.2 min (95% CI: 7.9, 20.5) per week between baseline and FU2. Sitting time decreased from baseline to FU2. Sitting time decreased with -1.4 h (95% CI: -1.7, -1.2) per day from baseline to FU2. Results for subgroup analyses are presented in Table 2.

**Table 2:** Mean and differences in physical activity and sitting time before and during the COVID-19 pandemic

	**All**^**1**^	**Women**^**2**^	**Men**^**2**^	** ≤ 25 years**^**3**^	** > 25 years**^**3**^
**Exercise**^**4**^					
Mean min/week at baseline	135	137	176	127	149
Mean difference in minutes (95% CI) from baseline to FU1	-5.7 (-10.0, -1.5)	-5.2 (-10.1, -0.2)	-7.7 (-16.0, 0.5)	-4.2 (-10.0, 1.6)	-7.7 (-13.9, -1.6)
Mean difference in minutes (95% CI) from baseline to FU2	-7.7 (-12.6, -2.8)	-6.9 (-12.7, -1.1)	-10.5 (-19.3, -1.7)	-5.5 (-11.9, 0.9)	-10.4 (-17.9, -2.9)
**Daily activities**^**5**^					
Mean min/week at baseline	154	143	154	139	154
Mean difference in minutes (95% CI) from baseline to FU1	5.6 (0.3, 11.7)	6.0 (-0.7, 12.6)	6.1 (-5.4, 17.5)	11.8 (4.3, 19.2)	-1.3 (-10.0, 7.5)
Mean difference in minutes (95% CI) from baseline to FU2	14.2 (7.9, 20.5)	14.3 (7.2, 21.4)	13.2 (-0.6, 26.9)	19.9 (11.3, 28.5)	7.4 (-1.9, 16.6)
**Total physical activity**					
Mean min/week at baseline	289	280	329	266	303
Mean difference in minutes (95% CI) from baseline to FU1	-0.2 (-7.7, 7.2)	0.3 (-8.4, 9.0)	-2.0 (-16.7, 12.7)	7.2 (-2.6, 17.0)	-9.5 (-20.9, 1.8)
Mean difference in minutes (95% CI) from baseline to FU2	6.0 (-2.3, 14.3)	6.9 (-2.7, 16.5)	2.2 (-14.7, 19.1)	13.9 (2.9, 25.0)	-3.7 (-16.2, 8.8)
**Sitting time**					
Mean hours/day at baseline	9.2	8.44	8.36	10.21	7.55
Mean difference in hours (95% CI) from baseline to FU1	-0.1 (-0.3, 0.1)	-0.1 (-0.4, 0.1)	0.2 (-0.2, 0.6)	-0.2 (-0.4, 0.1)	0.1 (-0.2, 0.3)
Mean difference in hours (95% CI) from baseline to FU2	-1.4 (-1.7, -1.2)	-1.5 (-1.8, -1.3)	-1.1 (-1.5, -0.6)	-1.7 (-2.0, -1.4)	-1.1 (-1.4, -0.8)

Abbreviations, FU1 follow-up 1 (March 14, 2020 to June 15, 2020), FU2 follow-up 2 (June 16, 2020 to September 10, 2020)

^1^Adjusted for follow-up period, age and sex

^2^Adjusted for follow-up period and age

^3^Adjusted for follow-up period and sex

^4^Exercise activities, i.e., running, fitness class, or ball games

^5^Activities that are not exercise, i.e., walks, bicycling, or gardening

Mean number of days per week of intake of each meal are presented in Table 3. Breakfast intake increased by 0.2 days (95% CI: 0.1, 0.3) between baseline and FU2 for the full group. Also, for morning snacks, there was a decrease of -0.2 days (95% CI: -0.2, -0.1) between baseline and FU1 and -0.4 days (95% CI: -0.5, -0.2) between baseline and FU2. Lunch intake decreased with -0.2 days (95% CI: -0.2, -0.1) between baseline and FU1 and -0.2 days (95% CI: -0.3, -0.0) between baseline and FU2. Afternoon snack decreased with -0.4 days (95% CI: -0.5, -0.2) between baseline and FU2, as well as dinner with -0.1 days (95% CI: -0.2, -0.0) between baseline and FU1 and -0.1 days (95% CI: -0.2, -0.0) between baseline and FU2. In contrary, intake of evening snack increased with 0.1 days (95% CI: 0.0, 0.3) between baseline and FU1. Results from subgroup analyses are presented in Table 3. Among irregular eaters at baseline (*n* = 1175), 1% reported they did so as they could not afford to eat regularly, 4% due to a special diet, 7% due to desire to lose weight, 21% due to lack of time, and 29% for other reasons. Proportion of answers were similar among participants answering this question (*n* = 763) at follow-up 2. Results for subgroup analyses are presented in Table 3.

**Table 3:** Meal frequency and differences before and during the COVID-19 pandemic

	**All**^**1**^	**Women**^**2**^	**Men**^**2**^	** ≤ 25 years**^**3**^	** > 25 years**^**3**^
**Breakfast**					
Mean days/week at baseline	4.4	5.6	5.0	4.2	5.2
Mean difference in days (95% CI) from baseline to FU1	0.1 (-0.0, 0.2)	0.1 (-0.0, 0.2)	-0.0 (-0.3, 0.2)	0.2 (0.0, 0.3)	-0.0 (-0.2, 0.2)
Mean difference in days (95% CI) from baseline to FU2	0.2 (0.1, 0.3)	0.1 (0.0, 0.3)	0.1 (-0.2, 0.4)	0.2 (0.0, 0.4)	0.1 (-0.0, 0.3)
**Morning snack**					
Mean days/week at baseline	2.0	2.5	2.1	2.0	2.0
Mean difference in days (95% CI) from baseline to FU1	-0.2 (-0.2, -0.1)	-0.3 (-0.5, -0.1)	-0.0 (-0.3, 0.2)	-0.2 (-0.4, -0.1)	-0.2 (-0.4, -0.0)
Mean difference in days (95% CI) from baseline to FU2	-0.4 (-0.5, -0.2)	-0.4 (-0.6, -0.2)	-0.2 (-0.5, 0.1)	-0.3 (-0.5, -0.1)	-0.4 (-0.6, -0.2)
**Lunch**					
Mean days/week at baseline	5.5	6.3	6.3	5.4	6.2
Mean difference in days (95% CI) from baseline to FU1	-0.2 (-0.2, -0.1)	-0.1 (-0.3, -0.0)	-0.2 (-0.3, 0.0)	-0.2 (-0.3, -0.1)	-0.1 (-0.2, 0.1)
Mean difference in days (95% CI) from baseline to FU2	-0.2 (-0.3, -0.0)	-0.2 (-0.3, -0.0)	-0.1 (-0.3, 0.1)	-0.2 (-0.3, -0.0)	-0.2 (-0.3, 0.0)
**Afternoon snack**					
Mean days/week at baseline	2.9	3.3	3.0	3.1	2.7
Mean difference in days (95% CI) from baseline to FU1	-0.1 (-0.2, 0.0)	-0.1 (-0.2, 0.1)	-0.2 (-0.5, 0.1)	-0.1 (-0.2, 0.1)	-0.1 (-0.3, 0.1)
Mean difference in days (95% CI) from baseline to FU2	-0.4 (-0.5, -0.2)	-0.4 (-0.5, -0.2)	-0.4 (-0.7, -0.1)	-0.5 (-0.7, -0.3)	-0.2 (-0.5, -0.0)
**Dinner**					
Mean days/week at baseline	6.3	6.6	6.7	6.3	6.7
Mean difference in days (95% CI) from baseline to FU1	-0.1 (-0.2, -0.0)	-0.1 (-0.2, -0.0)	-0.1 (-0.3, 0.1)	-0.1 (-0.2, 0.0)	-0.2 (-0.3, -0.1)
Mean difference in days (95% CI) from baseline to FU2	-0.1 (-0.2, -0.0)	-0.1 (-0.2, -0.0)	-0.1 (-0.3, 0.1)	-0.1 (-0.2, 0.0)	-0.2 (-0.3, -0.1)
**Evening snack**					
Mean days/week at baseline	3.1	2.7	3.2	3.1	2.7
Mean difference in days (95% CI) from baseline to FU1	0.1 (0.0, 0.3)	0.2 (0.0, 0.3)	0.1 (-0.2, 0.4)	0.1 (-0.1, 0.3)	0.2 (-0.0, 0.4)
Mean difference in days (95% CI) from baseline to FU2	0.1 (-0.0, 0.3)	0.2 (-0.0, 0.3)	0.1 (-0.2, 0.4)	0.1 (-0.1, 0.3)	0.2 (-0.0, 0.4)
**Other meal 1**					
Mean days/week at baseline	1.0	1.1	1.3	1.0	1.1
Mean difference in days (95% CI) from baseline to FU1	0.1 (-0.1, 0.2)	0.1 (-0.1, 0.2)	-0.0 (-0.3, 0.3)	-0.1 (-0.2, 0.1)	0.2 (-0.0, 0.4)
Mean difference in days (95% CI) from baseline to FU2	0.0 (-0.1, 0.2)	0.1 (-0.1, 0.2)	-0.1 (-0.4, 0.1)	-0.1 (-0.2, 0.1)	0.1 (-0.1, 0.3)
**Other meal 2**					
Mean days/week at baseline	0.5	0.6	0.7	0.5	0.5
Mean difference in days (95% CI) from baseline to FU1	0.2 (0.0, 0.3)	0.1 (0.0, 0.3)	0.2 (-0.1, 0.4)	0.1 (-0.1, 0.2)	0.2 (0.1, 0.4)
Mean difference in days (95% CI) from baseline to FU2	0.1 (-0.0, 0.2)	0.1 (-0.0, 0.2)	0.1 (-0.1, 0.4)	0.0 (-0.1, 0.2)	0.2 (-0.0, 0.3)

Abbreviations, FU1 follow-up 1 (March 14, 2020 to June 15, 2020), FU2 follow-up 2 (June 16, 2020 to September 10, 2020)

^1^Adjusted for follow-up period, age, and sex

^2^Adjusted for follow-up period and age

^3^Adjusted for follow-up period and sex

Mean ASSIST scores on the 42-point scale and changes in substance risk use are presented in Table 4. All mean values and changes in alcohol, tobacco and substance risk use were small between baseline and FU1 as well as between baseline and FU2 for the full group as well as for subgroups.

**Table 4:** Mean scores and changes in alcohol, tobacco, and substance use before and during the COVID-19 pandemic

	**All**^**1**^	**Women**^**2**^	**Men**^**2**^	** ≤ 25 years**^**3**^	** > 25 years**^**3**^
**Alcohol**					
Mean score at baseline	5.3	5.0	5.4	5.0	5.5
Mean difference in risk score (95% CI) from baseline to FU1	-0.8 (-1.0, -0.6)	-0.7 (-1.0, -0.5)	-1.1 (-1.2, -1.0)	-0.9 (-1.2, -0.6)	-0.6 (-1.0, -0.3)
Mean difference in risk score (95% CI) from baseline to FU2	-0.5 (-0.7, -0.2)	-0.4 (-0.7, -0.1)	-0.7 (-1.3, -0.1)	-0.5 (-0.9, -0.2)	-0.4 (-0.8, -0.1)
**Tobacco**					
Mean score at baseline	6.0	3.4	4.9	4.8	5.9
Mean difference in risk score (95% CI) from baseline to FU1	-0.4 (-0.6, -0.2)	-0.5 (-0.7, -0.2)	-0.2 (-0.6, 0.2)	-0.3 (-0.6, -0.1)	-0.5 (-0.8, -0.1)
Mean difference in risk score (95% CI) from baseline to FU2	-0.3 (-0.5, -0.1)	-0.4 (-0.7, -0.2)	0.1 (-0.3, 0.6)	0.0 (-0.2, 0.3)	-0.1 (-1.1, -0.3)
**Cannabis**					
Mean score at baseline	0.8	0.3	0.7	0.8	0.4
Mean difference in risk score (95% CI) from baseline to FU1	-0.0 (-0.1, 0.0)	-0.0 (-0.1, 0.1)	-0.1 (-0.3, 0.1)	-0.0 (-0.2, 0.1)	-0.1 (-0.2, -0.0)
Mean difference in risk score (95% CI) from baseline to FU2	-0.0 (-0.1, 0.1)	-0.0 (-0.1, 1.0)	-0.0 (-0.3, 0.3)	-0.0 (-0.3, 0.1)	0.0 (-0.1, 0.2)
**Cocaine**					
Mean score at baseline	0.1	0.1	0.2	0.1	0.2
Mean difference in risk score (95% CI) from baseline to FU1	-0.0 (-0.1, 0.0)	-0.0 (-0.0, 0.05)	-0.1 (-0.2, 0.0)	-0.0 (-0.1, 0.0)	-0.0 (-0.1, 0.1)
Mean difference in risk score (95% CI) from baseline to FU2	-0.0 (-0.1, 0.1)	0.0 (-0.1, 0.1)	-0.1 (-0.2, 0.0)	-0.0 (-0.1, 0.1)	-0.0 (-0.1, 1.0)
**Amphetamine**					
Mean score at baseline	0.3	0.1	0.1	0.2	0.4
Mean difference in risk score (95% CI) from baseline to FU1	-0.0 (-0.1, 0.0)	-0.0 (-0.1, 0.0)	-0.0 (-0.2, 0.1)	-0.0 (-0.1, 0.1)	-0.1 (-0.2, 0.0)
Mean difference in risk score (95% CI) from baseline to FU2	-0.0 (-0.1, 0.0)	-0.0 (-0.2, 0.0)	-0.0 (-0.1, 0.1)	0.0 (-0.0, 0.1)	-0.1 (-0.2, 0.0)
**Solvents**					
Mean score at baseline	0.0	0.0	0.1	0.0	0.0
Mean difference in risk score (95% CI) from baseline to FU1	-0.0 (-0.0, 0.0)	-0.0 (-0.0, 0.0)	-0.0 (-0.0, 0.0)	-0.0 (-0.1, 0.0)	-0.0 (-0.1, 0.0)
Mean difference in risk score (95% CI) from baseline to FU2	-0.0 (-0.0, 0.0)	-0.0 (-0.0, 0.0)	-0.0 (-0.1, 0.0)	-0.0 (-0.1, 0.0)	0.0 (-0.0, 0.1)
**Sedatives**					
Mean score at baseline	0.2	0.2	0.2	0.1	0.3
Mean difference in risk score (95% CI) from baseline to FU1	-0.1 (-0.2, -0.0)	-0.1 (-0.2, 0.0)	-0.1 (-0.3, 0.1)	-0.1 (-0.2, -0.0)	-0.1 (-0.2, 0.1)
Mean difference in risk score (95% CI) from baseline to FU2	-0.1 (-0.2, -0.0)	-0.1 (-0.2, 0.0)	-0.1 (-0.3, 0.1)	-0.1 (-0.2, -0.0)	-0.1 (-0.2, 0.0)
**Hallucinogens**					
Mean score at baseline	0.0	0.0	0.1	0.0	0.1
Mean difference in risk score (95% CI) from baseline to FU1	0.0 (-0.0, 0.0)	0.0 (-0.0, 0.0)	-0.0 (-0.1, 0.0)	-0.0 (-0.0, 0.0)	0.0 (-0.0, 0.0)
Mean difference in risk score (95% CI) from baseline to FU2	0.0 (-0.0, 0.0)	0.0 (-0.0, 0.0)	0.0 (-0.0, 0.1)	0.0 (-0.0, 0.0)	0.0 (-0.0, 0.1)
**Opiates**					
Mean score at baseline	0.0	0.1	0.0	0.0	0.1
Mean difference in risk score (95% CI) from baseline to FU1	-0.0 (-0.1, 0.0)	-0.0 (-0.1, 0.0)	-0.0 (-0.0, 0.0)	0.0 (-0.0, 0.0)	-0.1 (-0.1, -0.0)
Mean difference in risk score (95% CI) from baseline to FU2	-0.0 (-0.0, 0.0)	-0.0 (-0.1, 0.0)	0.0 (-0.0, 0.1)	-0.0 (-0.0, 0.0)	-0.0 (-0.1, 0.1)

Abbreviations, FU1 follow-up 1 (March 14, 2020 to June 15, 2020), FU2 follow-up 2 (June 16, 2020 to September 10, 2020)

^1^Adjusted for follow-up period, age, and sex

^2^Adjusted for follow-up period and age

^3^Adjusted for follow-up period and sex

### Sensitivity analyses

Results from the complete case GEE-analyses for total physical activity, sitting time, intake of breakfast, lunch, dinner as well as alcohol and tobacco use are presented in Additional file 1. The complete case analyses included 1150 participants. Only very small differences from the main results were observed regarding physical activity, breakfast intake and tobacco use, however, the same tendency in changes between periods of measurement was observed.

## Discussion

This study aimed to assess Swedish university students’ level of physical activity, sitting time, meal frequency, alcohol, tobacco, and illicit use of drugs before and during the COVID-19 pandemic. Our results show a slight improvement in these lifestyle behaviors during the pandemic compared to before; however, it is important to emphasize that changes between periods of measurement were small. Examining the long-term consequences of the COVID-19 pandemic on students’ lifestyle behaviors is warranted, given their substantial role for public health.

Time spent in daily activities increased and exercise and sitting time decreased. Most international studies report decreased levels of physical activity among students during the pandemic [23, 26, 28, 45–51], other studies report variations in behavior from unchanged [52] to increased [27, 53]. In a study by Jalal et al. (2021) of Saudi-Arabian university students’, there was an increase in sitting time from 449 min in March 2020 to 518 min in June 2020 [54]. Although sitting time among Swedish university students on average was higher during the same period in the current study, Jalal et al. reported a more substantial difference comparing behaviors before and during the pandemic [54]. Furthermore, the slightly increased physical activity in our study could be explained by that follow-up occurred during students’ summer break, when likely less time is spent sitting. Also, the spread of COVID-19 was less adverse in the summer and Swedish restrictions differed from full scale lock-down in other countries, enabling free movement in society.

Meal frequency changed to some extent during the study period. Intake of breakfast slightly increased over number of days from baseline to FU2, whereas number of days with lunch and dinner slightly decreased. These results conflict a previous study in which female university students reported skipping breakfast more often during the pandemic [55]. Furthermore, fewer morning snacks and afternoon snacks were reported, respectively, during the pandemic as compared to before. However, eating more other meals during the pandemic compared to before was more common, which could indicate a higher frequency of snacking outside main meals. These results are in line with international studies showing that snacking between meals and late at night have been more common during the pandemic both in general populations [56] and among students [28, 57]. Possible explanations could be due to boredom [58] and spending more time where food is easily accessible [28, 57]. Although food insecurity increased among students during the COVID-19 pandemic in international studies [32, 33], only 1% reported food irregularity due to economic constraints, hence, food insecurity is not considered an issue in this population.

Finally, our results show that students’ behaviors regarding risk use of alcohol, tobacco and illicit use of drugs changed to some extent during the pandemic. A slight decrease in risk alcohol consumption and use of tobacco was reported. It is important to stress that the use of alcohol, tobacco and drugs all correspond to low risk use according to the ASSIST scale (score 0–10 out of maximum score 42), and although some of the small changes were statistically significant regarding use of drugs, they are not considered clinically relevant. Previous studies report unchanged smoking habits among students during the pandemic [59], decreased use of tobacco [60] as well as no meaningful differences regarding use of tobacco, cannabis and alcohol [61]. Also increased consumption of alcohol and use of tobacco and cannabis during the pandemic [34] have been reported. Regarding alcohol, most studies involving university students report decreased consumption [37, 62–64] which could be explained by reduced social opportunities during confinement [63] and changed living situations [37, 64].

### Strengths and limitations

This prospective cohort study had a longitudinal design with a substantial sample size. All study participants were included prior to the implementation of remote learning in Swedish universities, March 17, 2020. This in turn enabled comparisons with the semester (from March 14, 2020) and summer break (from June, 16 2020) during the pandemic. This is a methodological advantage in comparison to many previous studies, which mainly have a cross-sectional approach where participants are asked to retrospectively report their health before the pandemic. Consequently, this introduces a risk of inaccurate conclusions about changes in lifestyle behaviors over time.

Another strength is the use of valid instruments for measuring lifestyle behaviors. However, self-reported health behaviors tend to be in accordance with social desirability [65] which, in this study, for example could be to underreport drug use and overreport physical activity levels. Also, there is a possibility of introducing recall bias [65].

The biggest threat to the validity is if attrition during the study period covary with the prevalence of unhealthy lifestyle behaviors at baseline. The follow-up rate between baseline and FU1 was 74%, and between baseline and FU2 was 62%. If the drop-out of study participants differ between those with healthy and unhealthy lifestyle behaviors, the conclusions could be inaccurate. Further, the fact that only 28% of the invited students participated may entail that students with more unhealthy behaviors were not included. To evaluate if the conclusions regarding changes in lifestyle over time are valid, a sensitivity analysis was performed. Only small differences were observed between the full group and those who completed all three measurements regarding total physical activity, breakfast, and tobacco use. The sensitivity analysis showed the same tendency as the main results (Additional file 1). Hence, the conclusions about changes in students’ lifestyle behaviors during the pandemic are considered valid.

Students were enrolled in educational programs in Stockholm County; hence, the sample should not be considered as representative of lifestyle behaviors among students in other parts of Sweden. Also, the external validity should be considered regarding that the majority were studying in the field of medicine and health sciences, and possibly could have better health literacy and lifestyle habits than students in general. For example, this is evident for total physical activity where average time spent in physical activity was 289 min per week, which is according to current recommendations [66].

## Conclusions

This study suggests that physical activity, sitting time, meal frequency, alcohol use, tobacco use, and illicit use of drugs in this sample of Swedish university students slightly improved during the first six months of the COVID-19 pandemic compared to before.

## Supplementary Information

**Additional file 1.** 

## Data Availability

The datasets generated and/or analyzed during the current study are not publicly available due to collection of sensitive personal information but are available from the corresponding author on reasonable request.

## Abbreviations

ASSIST: Alcohol, Smoking and Substance Involvement Screening Test
COVID-19: Coronavirus Disease 2019
FU1: Follow-up 1
FU2: Follow-up 2
GEE: Generalized Estimating Equations
SUN-study: Sustainable UNiversity Life study
