# Supplementary material for: Lifestyle behaviors in Swedish university students before and during the first six months of the COVID-19 pandemic: a cohort study
Source: BMC Public Health. 2022 Jun 16;22:1207. doi: 10.1186/s12889-022-13553-7 (PMC9202972; doi:10.1186/s12889-022-13553-7)
Supplement: Supplementary file 1 — Additional file 1. [file 12889_2022_13553_MOESM1_ESM.docx]

**Additional file 1.** Lifestyle behaviors before and during the COVID-19 pandemic for participants answering at all measurements (n=1150).

|  | | **All^1^** |
| --- | --- | --- |
| **Total physical activity^2^** | |  |
| Mean min/week at baseline | 295 |  |
| Mean difference in min (95% CI) from baseline to FU1 | -1.9 (-10.5, 6.7) |  |
| Mean difference in min (95% CI) from baseline to FU2 | 3.5 (-5.5, 12.5) |  |
| **Sitting time** | |  |
| Mean hours/day at baseline | 9.4 |  |
| Mean difference in hours (95% CI) from baseline to FU1 | -0.1 (-0.3, 0.2) |  |
| Mean difference in hours (95% CI) from baseline to FU2 | -1.5 (-1.7, -1.3) |  |
| **Breakfast** | |  |
| Mean days/week at baseline | 4.5 |  |
| Mean difference in days (95% CI) from baseline to FU1 | 0.0 (-0.1, 0.2) |  |
| Mean difference in days (95% CI) from baseline to FU2 | 0.1 (-0.0, 0.2) |  |
| **Lunch** | |  |
| Mean days/week at baseline | 5.6 |  |
| Mean difference in days (95% CI) from baseline to FU1 | -0.1 (-0.2, -0.0) |  |
| Mean difference in days (95% CI) from baseline to FU2 | -0.2 (-0.3, -0.1) |  |
| **Dinner** | |  |
| Mean days/week at baseline | 6.3 |  |
| Mean difference in days (95% CI) from baseline to FU1 | -0.1 (-0.2, -0.0) |  |
| Mean difference in days (95% CI) from baseline to FU2 | -0.1 (-0.2, -0.0) |  |
| **Alcohol** | |  |
| Mean risk score at baseline | 5.1 |  |
| Mean difference in risk score (95% CI) from baseline to FU1 | -0.7 (-0.9, -0.4) |  |
| Mean difference in risk score (95% CI) from baseline to FU2 | -0.4 (-0.7, -0.1) |  |
| **Tobacco** | |  |
| Mean risk score at baseline | 5.5 |  |
| Mean difference in risk score (95% CI) from baseline to FU1 | -0.3 (-0.6, -0.1) |  |
| Mean difference in risk score (95% CI) from baseline to FU2 | -0.2 (-0.5, 0.0) |  |

Abbreviations, FU1 follow-up 1 (March 14, 2020 to June 15, 2020), FU2 follow-up 2 (June 16, 2020 to September 10, 2020).
^1^Analyses are adjusted for period, age and sex.
^2^Combination of exercise activities, i.e., running, fitness class, or ball games and activities that are not exercise, i.e., walks, bicycling, or gardening.
